# Comparative Transcriptomics Reveals 129 Transcripts That Are Temporally Regulated during Anther Development and Meiotic Progression in Both Bread Wheat (*Triticum aestivum*) and Rice (*Oryza sativa*)

**DOI:** 10.1155/2011/931898

**Published:** 2011-10-17

**Authors:** Wayne Crismani, Sanjay Kapoor, Jason A. Able

**Affiliations:** ^1^School of Agriculture, Food & Wine, The Waite Research Institute, The University of Adelaide, Waite Campus, PMB1, Glen Osmond, SA 5064, Australia; ^2^Station de Génétique et Amélioration des Plantes, INRA, Centre de Versailles Grignon, Route de Saint-Cyr, 78026 Versailles, France; ^3^Interdisciplinary Center for Plant Genomics and Department of Plant Molecular Biology, University of Delhi, South Campus, Benito Juarez Road, New Delhi 110021, India

## Abstract

Meiosis is a specialised type of cell division in sexually reproducing organisms that generates genetic diversity and prevents chromosome doubling in successive generations. The last decade has seen forward and reverse genetic approaches identifying many genes in the plant kingdom which highlight similarities and differences in the mechanics of meiosis between taxonomic kingdoms. We present here a high throughput *in silico* analysis, using bread wheat and rice, which has generated a list of 129 transcripts containing genes with meiotic roles and some which are currently unknown.

## 1. Introduction

Since its inception over a decade ago, microarray technology has significantly increased its application-base and popularity. Initially developed to measure expression levels of given transcripts, microarrays provide a snapshot of the dynamic cellular transcriptomes which have been extracted from an isolated tissue-type. A common application of this technology is the comparison of the same tissue-type at the same stage of development between an experimental treatment or diseased tissue compared to a wild-type control. However, data from tissue time-courses/developmental series can also be generated with microarrays and have been reported in several species investigating different biological processes.

Meiosis is one such biological process and results in the formation of four genetically unique gametes, hence promoting genetic variation. Furthermore, meiosis is essential in sexually reproducing organisms as it prevents chromosome doubling in successive generations. Using microarray or SOLiD RNA-seq platforms, various studies have investigated the meiotic transcriptomes (often time-course experiments) in a variety of kingdoms. Examples include yeast (*Saccharomyces cerevisiae*) [1], *Drosophila* [2], *Caenorhabditis elegans* [3], rat (*Rattus rattus*) [4], mouse (*Mus musculus*) [5], bread wheat (*Triticum aestivum* L.) [6], and, more recently, rice (*Oryza sativa* L.) [7] and Arabidopsis (*Arabidopsis thaliana* L.) [8].

While our understanding of meiosis in some nonplant systems such as budding yeast is extensive, our knowledge of meiosis in plants is less advanced. Nonetheless, in the past 10 years (further to what has been achieved in Arabidopsis and rice), there has been an ongoing research effort towards building our knowledge across several different plant species, including barley (*Hordeum vulgare* L.) [9], wheat (*T. aestivum*) [10–12], maize (*Zea mays* L.) [13], and tomato (*Solanum lycopersicum* L.) [14]. With some exceptions (for example, where a gene has been plant-specific), such studies have concentrated on determining the function of one or two genes, that had been previously studied in nonplant systems, using reverse genetics. However, a study by Crismani et al. [6] detailing the first report investigating the meiotic transcriptome in any plant enabled analysis at a genome wide scale determining what genes were meiotically regulated across the extensive time-course examined. The plant in that study, bread wheat, is an allohexaploid with the genome being approximately 35 and 110 times the size of the rice and Arabidopsis genomes, respectively. Significantly, the Crismani et al. [6] study identified 142 transcripts (from a clustered subset of 350 transcripts) that were meiotically regulated but novel (when compared to all publically available sequence in the NCBI database at that time). More recently, in rice, the male gametophyte has also been examined using microarray technology [7]. This study identified a cluster of 372 transcripts that had a distinct meiotic-specific expression profile, from which 117 are either hypothetical/expressed or novel sequences with no annotations. Consequently, these two highlighted studies have facilitated the identification of many novel (and known) candidates that could be targeted for functional characterisation during meiosis in these species.

With these datasets being publically available, this short communication highlights that by comparatively analysing the wheat and rice meiotic transcriptomes, 129 transcripts that are common between these species during male gametophyte development have been identified. Further, expression analysis of 12 randomly selected transcripts (from the 129) between the two species revealed that seven had a correlation coefficient >0.6. Given the accessibility to rice mutant stocks and also putative homologues in Arabidopsis, this makes for an attractive approach in identifying the phenotype resulting from gene knockouts which would otherwise be a significant undertaking to achieve in bread wheat.

## 2. Materials and Methods

### 2.1. Microarray Datasets

Only two microarray datasets currently exist on the Gene Expression Omnibus database (http://www.ncbi.nlm.nih.gov/geo/), which represent an extensive time-course through male gametophyte development in cereals. The production of both the wheat [6] and rice [7] datasets has been described previously. The seven stages of wheat previously examined were premeiosis, leptotene to pachytene (LP), diplotene to anaphase I (DA), telophase I to telophase II (TT), tetrads (T), immature pollen (IP), and mature anthers (MAN) [6]. As the rice time-course material hybridised to the GeneChip Rice Genome Arrays was less detailed than the dataset from the wheat time-course, particular stages of the wheat dataset were excluded from the analysis or pooled, where appropriate. The four stages of male gametophyte development available for rice were premeiosis (PM), meiosis (M), single-celled pollen (SCP) otherwise herein referred to as immature pollen (IP) (comparable to the wheat IP stage), and trinucleate pollen (TPA) otherwise herein referred to as mature anthers (MAN) (comparable to the wheat MAN stage).

### 2.2. Data Reduction

The two datasets are very large with the wheat chip containing 60,703 probe sets and the rice chip 57,381 probe sets. To create a subset of transcripts enriched for potential meiotic transcripts, the two datasets were reduced significantly. For rice, *t*-tests were performed between PM and M from the microarray data to identify transcripts that were regulated by anther progression through meiosis. Probe sets were selected that had a corrected *P* value smaller than 0.05 between PM and M in addition to a log base 2 RMA-normalised value greater than five in at least one of the PM or M microarrays.

For wheat, as the previously reported microarray experiment separated meiotic stages specifically, data from a pool of material as broad as “meiotic” did not exist. To create a subset of data comparable to the rice PM versus M subset, *t*-tests were performed individually between the three PM replicates and the three replicates from the meiotic stages: LP, DA, and TT. The transcripts were then refined to only include those with a log base 2 RMA-normalised intensity greater than five in at least one of the microarrays hybridised with cRNA from the meiotic stages; PM, LP, DA, or TT. The results were then pooled. Therefore, transcripts which were expressed significantly different in more than one of the wheat *t*-tests were only included in the dataset once, thus creating a nonredundant dataset.

### 2.3. Sequence Retrieval, Further Data Filtration, and Transcript Annotation

The program—Fast tricks with FASTA—a useful bioinformatics tool (Dr. Ute Baumann, Australian Centre for Plant Functional Genomics, Adelaide, *unpublished data*) was used to retrieve the subset of sequences for the rice and wheat meiotically regulated transcripts from whole chip sequences. A database was created with the rice and the wheat subset sequences. To identify the transcripts within the wheat and rice subsets that shared strong sequence similarity (*E *value < *e* ^−30^) in addition to being meiotically regulated, Basic Local Alignment Search Tool (BLAST) analyses were performed between the two subsets of transcripts. The wheat and rice reduced datasets were reciprocally BLASTed against one another using both nucleotide BLAST (BLASTn) and a translated nucleotide BLAST (tBLASTx). The most similar hit was added to the further refined subsets of data for each query, given that they had occurred at a significance level below the set threshold. Transcripts which appeared as the most similar hit for more than one query were only included once. Annotations for the transcripts were retrieved from the NCBI database by using a batch BLAST program with a translated nucleotide database search using a translated nucleotide query (BLASTx) and tBLASTx to simultaneously identify annotated sequences (cutoff *E *value < *e* ^−20^).

### 2.4. Comparative Expression Profiling

The meiotically regulated data from the wheat and rice datasets was then centred by removing the average expression intensity value for a given transcript across their respective time-course. This removes the absolute values and replaces them with a movement about their average expression over the time-course with respect to doubling or halving their expression levels as the RMA-normalised data is presented as log base 2. This analysis places emphasis on expression trends across the time-course rather than absolute values. Hierarchical clustering was performed using a Euclidean squared similarity metric and an average linkage method (Acuity 4.0, Molecular Devices, Calif, USA). The expression profiles of 12 randomly selected transcripts from the final subset of 129 identified meiotically regulated and sequentially related transcripts between wheat and rice were then compared. In creating the pooled meiosis stage for wheat, the centred values for stages LP, DA, and TT were averaged.

## 3. Results

### 3.1. Data Filtration and Transcript Annotation

**Figure 1:**
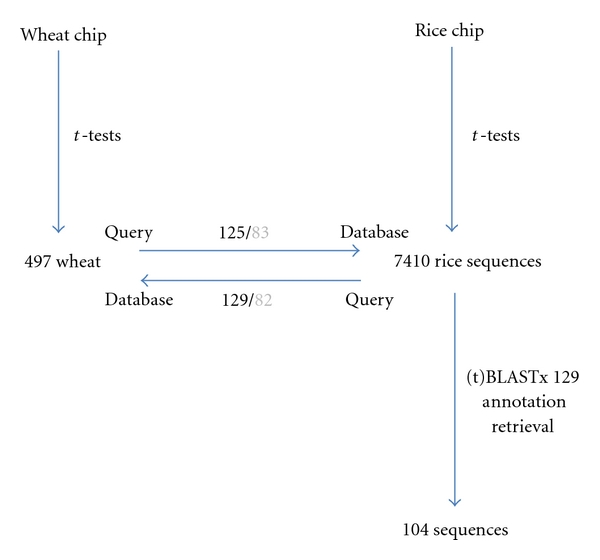
Schematic representing the data filtration that led to the identification of a subset of 129 meiotic transcripts from bread wheat and rice. *t*-tests identified transcripts which showed transient regulation during the development of anthers containing meiocytes. Reciprocal BLASTs were then performed between the wheat and rice subsets. tBLASTx results (black) and BLASTn results (grey) are shown. Functional annotations were retrieved from public databases using the 129 rice transcripts as the query. A total of 104 annotations were retrieved.

For the wheat analysis, PM versus LP resulted in no transcripts with a corrected *P* value equal to or smaller than 0.05. However, PM versus DA resulted in the identification of 415 transcripts while PM versus TT returned 181 transcripts. The union of these three sets of results revealed 497 nonredundant probe sets (Figure 1). Analysing the rice data with a *t*-test resulted in identifying 7,410 transcripts between the PM and M stages, which were regulated by the progression of anthers from PM to M. The reciprocal tBLASTx and BLASTn searches that were conducted between the two transcript subsets identified 83 sequences with BLASTn and 129 sequences with tBLASTx (Figure 1). Batch BLAST analysis of these 129 transcripts resulted in 104 annotations being retrieved where there was a putative ID associated with the sequence match (See Table S1 in Supplementary Material available online at doi: 10.1155/2011/931898). The remaining 25 transcripts were either not functionally annotated or returned hits below the accepted threshold (Table S1).

Based on the annotation (where available), all 129 transcripts were then assigned to a functional category (with “function not annotated” and “no hits found” also being classed as categories) (Table 1). The category with the highest number of representations was meiosis/cell division candidates, which accounted for 13.2% of the 129 transcripts (Table 1). Examples of these meiotic functions included a protein essential for synapsis of homologous chromosomes (ASY1) in bread wheat and Arabidopsis [11, 12, 15], a protein involved in signal transduction during the entry into meiosis in yeast (RIM11) [16], a gene involved in crossover formation (MLH3) [17], cell-cycle proteins, and chromosome morphogenesis genes (for example, multiple CDCs, a cyclin, and SPO76). The next highest, which also had the same number of transcripts as the meiosis/cell division category, was the function not annotated category (17 candidates). This category when combined with the transcripts where the set threshold was not reached (eight in total) collectively represents 19.4% of the 129 transcripts. 

Also of note was the category classified as transcription factors and nucleic acid binding, which included several proteins that are broadly defined as zinc fingers (Table 1, Table S1). The category classifications of the remaining 74 transcripts included but were not limited to cellular, secondary, and lipid metabolism through to biotic and abiotic stress related annotations (Table 1, Table S1).

### 3.2. Comparative Expression Profiling

**Figure 2:**
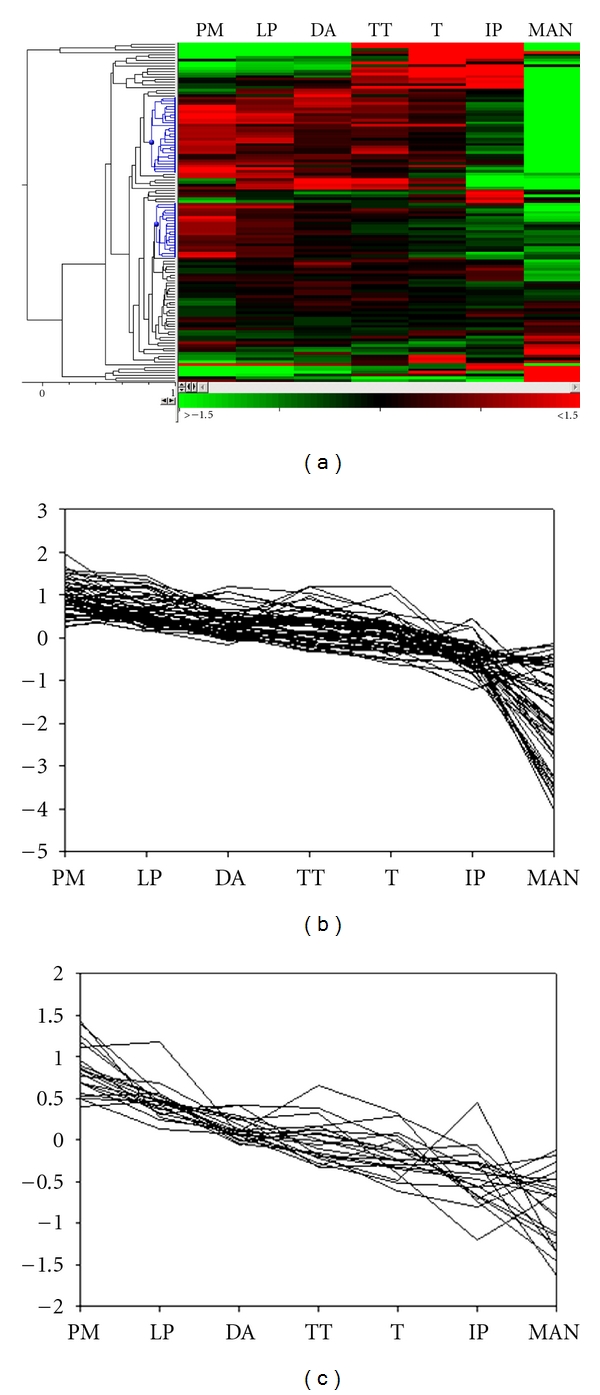
Hierarchical clustering of 125 wheat transcripts that were regulated over the progression of meiosis. The expression profiles of 125 transcripts (rows) were grouped across seven anther stages (columns) in a heat map (a). Similar expression profiles are clustered together as indicated by the dendrogram. The expression profiles of two clusters that are preferentially expressed in early meiosis which display similar expression profiles are highlighted in blue on the dendrogram. These clusters representing 28 and 20 transcripts are also shown separately in (b) and (c), respectively. Premeiosis (PM), leptotene–pachytene (LP), diplotene–anaphase I (DA), telophase I–telophase II (TT), tetrads (T), immature pollen (IP), mature anthers (MAN). Expression values (indicated by green through to red in colour) are centred, log base 2, RMA-normalised values.

**Figure 3:**
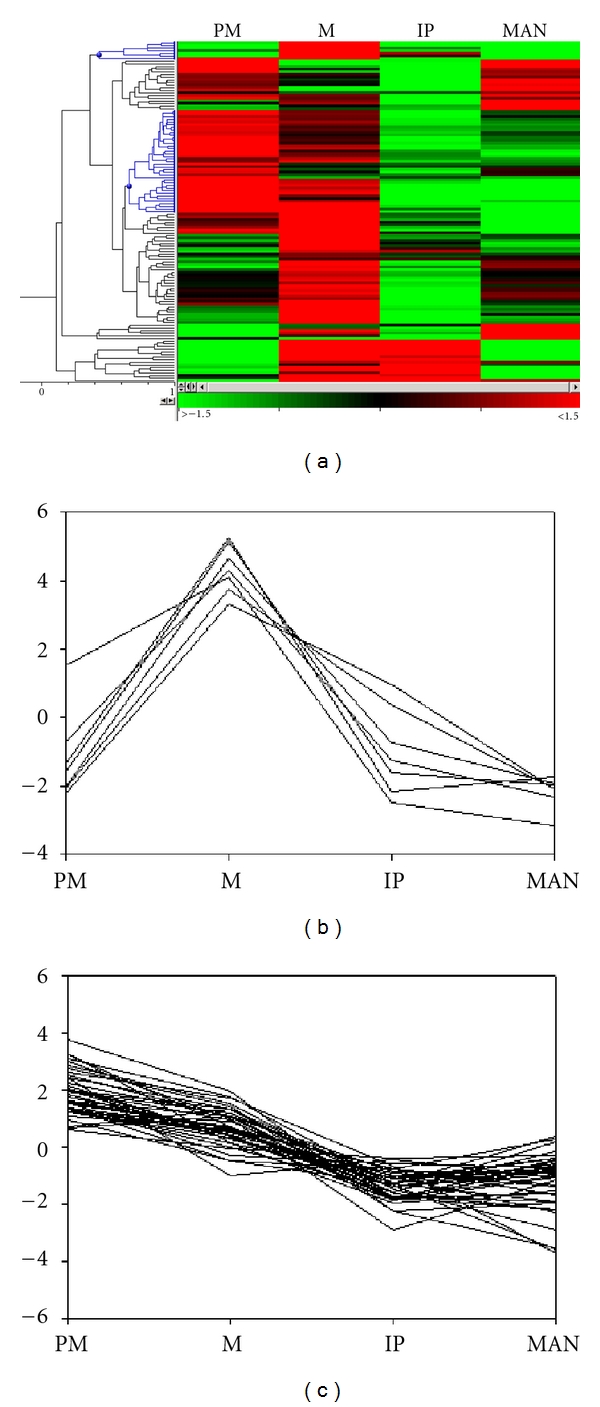
Hierarchical clustering of 129 rice transcripts that were regulated over the progression of meiosis. The expression profiles of 129 transcripts (rows) were grouped across four anther stages (columns) in a heat map (a). Similar expression profiles are clustered together as indicated by the dendrogram. The expression profiles of two clusters that are preferentially expressed in premeiotic and/or meiotic stages which display similar expression profiles are highlighted in blue on the dendrogram. These clusters representing 7 and 39 transcripts are also shown separately in (b) and (c), respectively. Premeiosis (PM), meiosis (M), immature pollen (IP), mature anthers (MAN). Expression values (indicated by green through to red in colour) are centred, log base 2, RMA-normalised values.

Transcripts with similar expression profiles were clustered together using hierarchical clustering using both the wheat (125) and rice (129) transcript datasets (Figures 2 and 3). This resulted in the identification of a number of interesting clusters in wheat (Figure 2). A group of 28 transcripts from the wheat dataset were expressed at higher levels during the majority of meiosis when compared to the other stages examined (Figure 2(b)). Several of these transcripts showed strong sequence similarities to histones and chromatin remodelling factors, proteins controlling cell cycle, recombination, and synapsis. Another cluster of interest with 20 transcripts was also expressed preferentially in premeiosis but was downregulated at a greater rate than the cluster aforementioned (Figure 2(c)). This cluster contained putative homologues of proteins involved in crossover formation, cell division, microtubule function, and chromatin remodelling.

Similarly, two distinct clusters were observed from the rice dataset (Figure 3). Indeed, these clusters (totalling 46 transcripts) were even more pronounced in rice (Figures 3(b) and 3(c)). However, the annotations of these 46 transcripts from rice identified fewer transcripts that can easily be associated with meiotic functions when compared to the wheat transcripts. The first distinct rice cluster, which was represented by only seven transcripts, contained *OsPAIR2* (essential for synapsis in rice [18]), putative homologues of a PIWI domain containing protein (germline-specific RNAi components), and a cyclin (Figure 3(b)). Within the second rice cluster, 39 transcripts showed higher levels of expression during the premeiotic and meiotic stages and then lower transcript levels in the remaining two stages of IP and MAN (Figure 3(c)). Some of these rice transcripts showed similarity to genes that have roles in chromatin remodelling.

**Figure 4:**
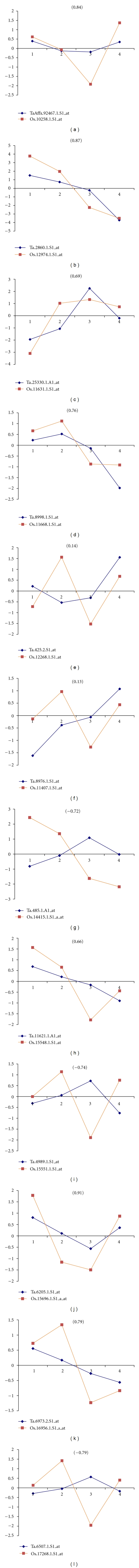
The correlation of 12 randomly selected, meiotically regulated expression profiles identified from the wheat and rice datasets. Blue diamonds and orange squares represent the wheat and rice transcripts, respectively. Numbers in parentheses represent the correlation coefficient between the two expression profiles. Expression values on the *Y*-axes are centred, log base 2, RMA-normalised values. Stages of anther development are displayed on the *X*-axes as 1 to 4, which represent premeiosis, meiosis, immature pollen, and mature anthers, respectively.

To determine whether a relationship between the expression profiles of selected transcripts from wheat and rice existed, 12 randomly selected transcripts from the dataset of 129 meiotically regulated and sequentially related transcripts were compared. While the correlation coefficient values between the wheat and rice expression profiles varied for these 12 transcripts, both a positive correlation as high as 0.91 and a moderate-high negative correlation of −0.79 were recorded. In total, seven out of the 12 transcripts shared a correlation coefficient stronger than 0.6 (Figure 4).

To extend the utility of this study outside of two important cereal species, we also identified putative homologues in Arabidopsis and Poplar (*Populus trichocarpa *Torr. & A. Gray) for a select number of transcripts from wheat and rice. An electronic fluorescent pictograph (eFP) browser (http://bbc.botany.utoronto.ca/efp/cgi-bin/efpWeb.cgi/) was then used to provide an indication as to whether the transcripts showed preferential expression in meiotic material in either of these additional plant species. Several transcripts (including *ASY1*) showed preferential expression in reproductive organs (buds in Arabidopsis and catkins in *P. trichocarpa*). For example, primary gene ID numbers including *At1g01280*, *At1g02050*, and *At1g67370* (*ASY1*) show preferential expression in Arabidopsis buds at or near meiosis. Similar results are seen in male meiotic tissue (male catkins) of the respective putative *P. trichocarpa* homologues (*Ptpaffx.202268.1.S1_at*, *Ptpaffx.202128.1.S1_at* and *Ptpaffx.153910.1.a1_at*, resp.). However, numerous transcripts showed patterns with stronger expression in vegetative tissues (e.g., primary gene ID numbers; *At1g05010* and the respective Poplar homologue, *Ptp.5158.1.S1_at*).

## 4. Discussion

### 4.1. Data Filtration

The wheat and rice data filtration method used to identify transcripts putatively having meiotic roles resulted in very different numbers being obtained; 497 and 7,410, respectively. As previously mentioned, the wheat genome has not yet been sequenced, and while the genome size varies considerably between these two species and the gene number will also be variable, the large observed difference must be accounted to something else. Most likely, this is due to two independent research groups being responsible for the harvesting and staging of collected wheat and rice anthers that were subsequently used in the array experiments previously reported [6, 7]. *t*-tests between the meiotic tissues and IP or MAN were excluded from the analysis as IP and MAN have very different profiles at the transcript level with the vast majority of the genome being temporally regulated during these stages. Including these stages would have resulted in an overestimation of probe sets that are involved in meiosis during the development of the male gametophyte.

The reciprocal BLASTs between the 497 and 7,410 were expected to return approximately the same number of hits as the majority of genes in rice would have a wheat homologue based on both sequence and function. This was found to be the case with 125/83 returned for wheat to rice, while 129/82 were returned for rice to wheat. However, microarrays can contain probe sets designed against multiple noncontiguous ESTs representing unique parts of a whole transcript from one species. In the case where the homologous transcript in the other species is represented by a full-length sequence in the database, a discrepancy will occur when using a reciprocal BLAST. This becomes increasingly likely given that the wheat chip was designed as a “discovery chip” from the wheat ESTs which were present in the public databases in 2004. The rice chip is a much closer representation of what is the entire transcriptome in this plant species. Generally, however, there was only a discrepancy of one significant hit using BLASTn or four significant hits when using the tBLASTx program (Figure 1).

104 transcripts shared similarity with previously annotated sequences. The most common annotation was meiotic/cell-cycle. The two processes were pooled since they are very difficult to uncouple as meiosis is a specialised type of cell cycle division. There are close to 50 meiotic genes in plants that have been identified and characterised to date [19], and the categorical data presented here included only two of these 50 or so meiotic genes,* ASY1* and *MLH3*, in addition to various proteins related to cell cycle and chromatin remodelling. The inability to detect a higher percentage of the 50 known meiotic genes suggests that, in wheat and rice at least, (1) the remaining known genes not identified by this method have a more static expression profile than the transcripts that matched the selection criteria used in this study; (2) they were not present on the wheat chip when developed and therefore were not detected; and/or (3) some of the sequences have not been conserved across species.

Significantly, from a gene discovery perspective, there were 25 transcripts with no similarity to functionally characterised genes which potentially have roles as important to meiosis as *ASY1*, *MLH3*, and chromatin remodelling. Other annotations such as roles in embryo development and tapetal-specific roles are consistent with the type of background expression profiles detected in a meiotic time-course, especially as whole anthers were used in both the wheat and rice experiments [6, 7].

### 4.2. Comparative Expression Profiling

Hierarchical clustering of the wheat and rice data subsets grouped transcripts with similar expression profiles adjacent to one another in the dendrogram. When comparing species, there were similarities between the results produced in the heat map clusters for wheat and rice despite the lower resolution of the rice data set. Analysis of the clusters which are preferentially expressed during meiosis did reveal an enrichment of meiotic/cell-cycle transcripts. Given that there are approximately 50 known meiotic genes in plants and approximately 41,000 genes predicted to be in the rice genome (excluding transposable element related genes—see http://rice.plantbiology.msu.edu), this represents 0.12% of the genome. Therefore, the identification of *ASY1*, *MLH3*, and *SPO76* alone in the wheat and rice datasets is approximately a 19-fold increase in meiotic transcript enrichment than the known genomic average.

The comparison of 12 randomly selected wheat/rice transcripts from the subset of 129 revealed that the expression profiles between more than half of these shared a correlation coefficient greater than 0.6. This suggests that not all putative homologues are expressed identically from the time they diverged over the course of evolution. Nonetheless, there are still common themes in the grasses and by analysing sequences across several species, this has been shown for many important genes involved in early meiosis [20]. Other factors that must be taken into consideration when considering the level of correlation that has been observed between the wheat and rice datasets investigated in this study include the variation in staging between the species and also the environmental conditions in which the plants were grown before anther harvesting. Both of these, in addition to other factors, may have influenced the expression of the individual transcripts analysed.

The cross-species expression analysis was further extended to include Arabidopsis and *P. trichocarpa* by using the eFP browser tool [21]. Analysis of putative Arabidopsis and *P. trichocarpa* homologues from selected wheat and rice transcripts in the eFP browser revealed some commonalities between all four plant species. Such a finding suggests that the information generated from wheat and rice is transferrable not only reciprocally but also across from monocotyledonous plants to dicotyledons (where sequence and expression data exists). This infers that the results of whole genome screens in one plant species can be used as a guide for screening meiotic mutants in other plant species. In concluding, we propose that this list of 129 transcripts, with particular focus on the 25 novel transcripts, form the basis of a reverse genetic screen for identifying genes involved in plant meiosis.

## Supplementary Material

Table S1 highlights the BLASTx results using 129 rice sequences as the queries against the non-redundant database. The annotations of the 129 rice sequences also represent the 125 wheat sequences.

## Figures and Tables

**Table 1:** Biological classifications for 129 meiotically regulated wheat and rice transcripts. Annotations retrieved from NCBI were functionally categorised by manually searching the available literature. Numbers in parentheses correspond to the percentage representation within the 129 transcripts. No hits found imply so for a threshold *E*-value < *e* ^−20^. Percentage representations have been rounded-up to one decimal place.

Category	Representations (%)
Meiosis/cell cycle	17 (13.2)
Transcription factors and nucleic acid binding	13 (10.1)
Cellular metabolism	12 (9.3)
Organelle activity	10 (7.8)
Biotic stress-related	9 (7.0)
Signal transduction	8 (6.2)
Secondary metabolism	6 (4.7)
Protein metabolism	6 (4.7)
Membrane transport	5 (3.9)
Hormone regulation	4 (3.1)
Protein transport	3 (2.3)
Abiotic stress response	2 (1.6)
Cell wall-related	2 (1.6)
Lipid metabolism	2 (1.6)
Tapetal function	1 (0.8)
Protein folding	1 (0.8)
Embryonic development	1 (0.8)
Ribosomal	1 (0.8)
Development	1 (0.8)
Function not annotated	17 (13.2)
No hits found	8 (6.2)
