# Supplementary material for: Comparative Transcriptomics Reveals 129 Transcripts That Are Temporally Regulated during Anther Development and Meiotic Progression in Both Bread Wheat (Triticum aestivum) and Rice (Oryza sativa)
Source: Int J Plant Genomics. 2011 Oct 17;2011:931898. doi: 10.1155/2011/931898 (PMC3199073; doi:10.1155/2011/931898)
Supplement: Supplementary file 1 — Table S1 highlights the BLASTx results using 129 rice sequences as the queries against the non-redundant database. The annotations of the 129 rice sequences also represent the 125 wheat sequences. [file 931898.f1.pdf]

Supplementary Material

Table S1. BLASTx results using 129 rice sequences as the queries against the non-redundant database. The annotations of the 129 rice sequences also represent the 125 wheat sequences.

Blast Search Results: database: nr

QUERY	Length (bp)	Database match	EXP	Putative ID
consensus:Rice:OsAffx.19544.1.S1_x_at; tigr|9640.m00715;	2973	ref|NP_001065922.1|	0	dehydration-responsive protein-related
consensus:Rice:Os.20348.1.S1_at; gb|AK066417.1;	2581	ref|NP_001057376.1|	0	NIK1 (NSP-INTERACTING KINASE 1
consensus:Rice:OsAffx.3315.1.S1_s_at; tigr|9631.m02431;	1509	ref|NP_001060447.1|	7.00E-83	unknown protein
consensus:Rice:Os.54526.1.S1_at; gb|AK105520.1;	1450	ref|NP_001049387.1|	2.00E-96	polygalacturonase, putative / pectinase
consensus:Rice:Os.2319.1.S2_at; gb|AF134807.2;	3922	ref|NP_001063545.1|	e-111	dihydroflavonol 4-reductase family / dihydrokaempferol 4-reductase family
consensus:Rice:Os.6101.1.S1_at; gb|AK106302.1;	1842	ref|NP_001052244.1|	7.00E-87	Probable UDP-glucosyl transferase
consensus:Rice:Os.35425.1.S1_at; gb|AK067556.1;	2624	ref|NP_001044732.1|	0	ATPase, coupled to transmembrane movement of substances
consensus:Rice:OsAffx.28544.2.S1_x_at; tigr|9635.m02165;	1290	ref|NP_001059449.1|	e-132	chalcone synthase family protein
consensus:Rice:Os.33605.2.S1_x_at; gb|AK069643.1;	1452	ref|NP_001043999.1|	3.00E-22	zinc finger (C3HC4-type RING finger) family protein
consensus:Rice:Os.55120.1.S1_at; gb|AK107521.1;	1750	ref|NP_001053872.1|	2.00E-91	PAZ domain-containing protein / piwi domain-containing protein

consensus:Rice:Os.17268.1.S1_at; gb|AK104878.1;	1354	ref|NP_001063624.1|	6.00E-99	putative peroxisomal membrane carrier protein
consensus:Rice:OsAffx.16813.1.S1_x_at; tigr|9636.m00286;	3498	ref|NP_001060926.1|	0	CYP703/CYP703A2 (CYTOCHROME P450, FAMILY 703, SUBFAMILY A, POLYPEPTIDE 2)
consensus:Rice:OsAffx.16983.1.S1_at; tigr|9636.m01363;	1002	no link		No hits above cut off
consensus:Rice:Os.52786.1.S1_at; gb|AK069250.1;	1361	ref|NP_001062210.1|	2.00E-60	unnamed protein product
consensus:Rice:OsAffx.28096.1.S1_at; tigr|9634.m04281;	1926	ref|NP_001058232.1|	e-166	unknown protein
consensus:Rice:Os.9496.1.S1_at; gb|AK064038.1;	1307	ref|NP_001065487.1|	7.00E-51	cinnamoyl-CoA reductase-related
consensus:Rice:Os.19331.1.S1_at; gb|AK071286.1;	1610	ref|NP_001055921.1|	1.00E-120	MtN21 nodulin protein-like
consensus:Rice:Os.34647.1.S1_at; gb|AK070644.1;	2176	ref|NP_001056404.1|	0	MAP kinase
consensus:Rice:Os.54800.1.S1_at; gb|AK106792.1;	2366	ref|NP_001058033.1|	0	ABC transporter family protein
consensus:Rice:Os.52091.1.S1_at; gb|AK064698.1;	1807	ref|NP_974827.1|	e-127	pectinacetylesterase family protein
consensus:Rice:Os.11558.1.S1_at; gb|AK101508.1;	1620	ref|NP_001042703.1|	1.00E-60	peroxidase, putative
consensus:Rice:Os.56988.1.S1_at; gb|AK110695.1;	952	ref|NP_001051269.1|	5.00E-50	minor allergen
consensus:Rice:Os.2550.1.S1_at; gb|AK109476.1;	2165	ref|NP_001064930.1|	4.00E-72	unknown protein
consensus:Rice:Os.15696.1.S1_a_at; gb|AB081464.1;	1901	ref|NP_001047562.1|	e-179	BETA-VPE (vacuolar processing enzyme beta); cysteine-type endopeptidase
consensus:Rice:Os.9955.1.S1_a_at; gb|AK068737.1;	1914	ref|NP_001042602.1|	0	ATSK11 (Arabidopsis thaliana SHAGGY-related kinase 11);
consensus:Rice:OsAffx.7832.1.S1_at; tigr|9640.m03777;	1521	ref|NP_001067112.1|	1.00E-51	unknown protein
consensus:Rice:Os.46304.1.S1_a_at; gb|CB668496;	837	no link		No hits above cut off

consensus:Rice:Os.54831.1.S1_at; gb|AK106857.1;	789	no link		No hits above cut off
consensus:Rice:OsAffx.29643.1.S1_at; tigr|9636.m04407;	429	ref|NP_001062420.1|	1.00E-10	A9 protein precursor-like
consensus:Rice:Os.35089.1.S1_at; gb|AK069013.1;	1262	ref|NP_001053131.1|	6.00E-48	CDC48-interacting UBX-domain protein
consensus:Rice:Os.56156.1.S1_at; gb|AK109467.1;	1597	ref|NP_001051964.1|	e-116	MATE efflux family protein
consensus:Rice:Os.31518.1.S1_at; gb|AK107209.1;	1725	ref|NP_001042758.1|	0	Cyclin-B1-3 (G2/mitotic-specific cyclin-B1-3)
consensus:Rice:OsAffx.13955.1.S1_x_at; tigr|9632.m02290;	1668	ref|NP_001052437.1|	e-174	4-coumarate--CoA ligase family protein / 4-coumaroyl-CoA synthase family protein
consensus:Rice:Os.10860.2.S1_at; gb|AK069437.1;	2127	ref|NP_001053351.1|	0	YSL7 (YELLOW STRIPE LIKE 7)
consensus:Rice:Os.45920.1.S1_at; gb|CR281284;	1720	ref|NP_001048679.1|	0	tubulin beta-8
consensus:Rice:Os.54989.1.S1_at; gb|AK107258.1;	1620	ref|NP_001062795.1|	4.00E-88	UBIQUITIN-CONJUGATING ENZYME 25
consensus:Rice:Os.5124.1.S1_at; gb|AK070411.1;	2540	ref|NP_001056382.1|	0	EMBRYO DEFECTIVE 2742
consensus:Rice:Os.16882.1.S1_at; gb|AK121304.1;	3305	ref|NP_001061466.1|	1.00E-47	peptidyl-prolyl cis-trans isomerase
consensus:Rice:OsAffx.27333.1.S1_at; tigr|9633.m04302;	1059	ref|NP_001044073.1|	e-128	choline kinase, putative
consensus:Rice:Os.5873.1.S1_at; gb|AK100301.1;	1523	ref|NP_001053779.1|	2.00E-34	Pspzf zinc finger protein-like
consensus:Rice:Os.12268.1.S1_at; gb|AF049889.1;	3119	ref|NP_001048259.1|	e-117	ethylene-forming enzyme
consensus:Rice:OsAffx.17435.1.S1_at; gb|NM_188999.1;	1035	ref|NP_001062236.1|	e-111	dihydroflavonol 4-reductase family / dihydrokaempferol 4-reductase family
consensus:Rice:Os.470.1.S1_s_at; gb|AK121722.1;	4839	ref|NP_001043541.1|	0	PLEIOTROPIC DRUG RESISTANCE 12
consensus:Rice:Os.11107.1.S1_at; gb|AK100744.1;	1890	ref|NP_171649.1|	2.00E-97	UDP-glucoronosyl/UDP-glucosyl transferase family protein

consensus:Rice:Os.13973.3.S1_at; gb|AK060575.1;	1931	ref|NP_001055303.1|	e-108	protein phosphatase 2C, putative
consensus:Rice:Os.3422.1.S1_at; gb|AK063598.1;	1711	ref|NP_001051822.1|	0	actin 7
consensus:Rice:Os.46383.2.A1_s_at; gb|CB652033;	838	ref|NP_001047825.1|	2.00E-68	phosphoribulokinase precursor
consensus:Rice:Os.8511.1.S1_s_at; gb|AK100972.1;	1744	ref|NP_001051991.1|	2.00E-91	cytochrome P450, family 93
consensus:Rice:Os.28096.1.S1_at; gb|AK060834.1;	1759	ref|NP_001055991.1|	e-155	CALRETICULIN 3; calcium ion binding
consensus:Rice:Os.38001.1.A1_at; gb|AK073434.1;	2291	ref|NP_001048588.1|	e-133	leucine-rich repeat family protein
consensus:Rice:Os.11219.1.S1_at; gb|BP184466;	1744	ref|NP_001044754.1|	2.00E-42	Histone H2B
consensus:Rice:Os.46325.1.A1_at; gb|AK120658.1;	1947	ref|NP_001042129.1|	e-147	zinc finger (C3HC4-type RING finger) family
consensus:Rice:Os.4731.1.A1_s_at; gb|CB661833;	1358	gb|EAY96137.1|	1.00E-50	unknown protein
consensus:Rice:Os.18877.1.S1_at; gb|AK060336.1;	1167	ref|NP_001055547.1|	e-109	ISOPENTENYL DIPHOSPHATE ISOMERASE 1
consensus:Rice:Os.15571.1.S1_at; gb|AK063753.1;	1482	ref|NP_001067285.1|	e-159	pyruvate dehydrogenase E1 beta subunit
consensus:Rice:Os.9110.1.S1_at; gb|AK071305.1;	1755	ref|NP_001063681.1|	e-160	thioesterase family protein
consensus:Rice:Os.18206.1.S1_at; gb|AK062929.1;	635	ref|NP_001044595.1|	3.00E-43	60S ribosomal protein L30
consensus:Rice:OsAffx.15017.1.S1_at; tigr|9633.m03610;	2094	ref|NP_187577.1|	0	minichromosome maintenance (MCM8/REC) protein
consensus:Rice:Os.2241.1.S1_a_at; gb|AK059818.1;	1184	ref|NP_001044339.1|	5.00E-36	Glutathione S-transferase
consensus:Rice:Os.17881.1.S1_at; gb|AK100467.1;	2537	ref|NP_001062612.1|	0	coatomer protein complex, subunit alpha, putative
consensus:Rice:Os.18583.1.S1_at; gb|AK073783.1;	2092	ref|NP_001042118.1|	e-116	hypothetical protein
consensus:Rice:Os.14415.1.S1_a_at; gb|AK072689.1;	3169	ref|NP_001050995.1|	0	LOX5; lipoxygenase

consensus:Rice:Os.21075.1.S1_at; gb|AK110878.1;	1982	ref|NP_001066285.1|	e-179	putative growth regulator protein
consensus:Rice:Os.53929.1.S1_at; gb|AK100884.1;	2053	ref|NP_001055220.1|	0	NADH dehydrogenase
consensus:Rice:Os.4421.1.S1_at; gb|AK102494.1;	2226	ref|NP_001056550.1|	0	SKU5 (skewed 5); copper ion binding
consensus:Rice:Os.11631.1.S1_at; gb|AK120783.1;	2021	ref|NP_001057353.1|	e-113	aspartyl protease family protein
consensus:Rice:OsAffx.17580.1.S1_at; tigr|9637.m00423;	1266	ref|NP_001062648.1|	e-138	CDC20.1; signal transducer
consensus:Rice:Os.372.1.S1_a_at; gb|AY078072.1;	1961	ref|NP_001053132.1|	0	glucose-6-phosphate 1-dehydrogenase
consensus:Rice:Os.28409.5.S1_at; gb|AK104167.1;	1224	no link	9.00E-179	Arabidopsis thaliana chloroplast DNA
consensus:Rice:Os.38299.1.S1_at; gb|AK102668.1;	1461	ref|NP_001058750.1|	3.00E-100	protein phosphatase 2C family protein
consensus:Rice:Os.49778.1.S1_at; gb|AB109238.1;	2180	ref|NP_001063610.1|	e-174	Asynapsis 1 (ASY1)
consensus:Rice:Os.18189.1.S1_at; gb|AK073256.1;	1278	ref|NP_001047974.1|	1.00E-71	Chromatin structure-remodeling complex protein BSH (Protein bushy) - (SNF5 homolog)
consensus:Rice:Os.18395.1.S1_s_at; gb|AK062516.1;	769	ref|NP_001057346.1|	1.00E-27	gibberellin-responsive protein, putative
consensus:Rice:Os.8647.1.S1_at; gb|AK120851.1;	2630	ref|NP_001046976.1|	2.00E-35	unknown protein
consensus:Rice:Os.51150.1.S1_s_at; gb|AK061163.1;	1329	ref|NP_001058014.1|	4.00E-44	putative ethylene responsive element
consensus:Rice:OsAffx.24019.1.S1_x_at; tigr|9629.m07214;	3426	ref|NP_001045457.1|	0	ATMLH1 (ARABIDOPSIS THALIANA MUTL-HOMOLOGUE 1)
consensus:Rice:OsAffx.11847.1.S1_at; tigr|9629.m07373;	831	ref|NP_001054459.1|	e-104	inorganic diphosphatase/ pyrophosphatase
consensus:Rice:Os.35685.1.S1_at; gb|AK066300.1;	2681	ref|NP_001044387.1|	0	glycosyl hydrolase family 3 protein
consensus:Rice:Os.15548.1.S1_at; gb|AK111670.1;	1752	ref|NP_001061119.1|	e-170	transducin family protein / WD-40 repeat family protein

consensus:Rice:Os.27371.1.A1_at; gb|AK065263.1;	2052	ref|NP_001056967.1|	e-171	beta-glucuronidase
consensus:Rice:Os.15551.1.S1_at; gb|AK059480.1;	1369	gb|AAP88334.1|	3.00E-78	unknown protein
consensus:Rice:OsAffx.15881.1.S1_at; tigr|9634.m04026;	2178	ref|NP_001058099.1|	0	HOBBIT (cell division cycle protein 27)
consensus:Rice:Os.49195.1.S1_at; gb|AK103138.1;	2084	ref|NP_001049413.1|	0	glycosyl hydrolase family 17 protein / beta-1,3-glucanase, putative
consensus:Rice:Os.54997.1.S1_at; gb|AK107276.1;	947	ref|NP_001051116.1|		No hits above cut off
consensus:Rice:Os.5187.1.S1_at; gb|AK062162.1;	1115	ref|NP_001060690.1|	2.00E-59	unknown protein
consensus:Rice:Os.27969.1.S1_at; gb|AK064894.1;	890	ref|NP_001057309.1|	7.00E-35	Nudix hydrolase 21, chloroplast precursor
consensus:Rice:Os.53771.1.A1_at; gb|AK099930.1;	4185	ref|NP_001062645.1|	0	nucleotide binding
consensus:Rice:Os.16956.1.S1_s_at; gb|AK103608.1;	2859	ref|NP_001043256.1|	0	early-responsive to dehydration protein-related / ERD protein-related
consensus:Rice:Os.54158.1.S1_at; gb|AK102230.1;	2844	ref|NP_001056445.1|	e-158	putative transposase
consensus:Rice:Os.9121.1.S1_at; gb|AK073973.1;	2957	ref|NP_199003.1|	e-109	POLA3/POLA4; DNA primase
consensus:Rice:Os.8979.1.S1_at; gb|AK111523.1;	1914	ref|NP_001064441.1|	e-143	kelch repeat-containing F-box family protein
consensus:Rice:Os.51962.1.S1_at; gb|AK064417.1;	2399	ref|NP_001064187.1|	8.00E-84	unknown protein
consensus:Rice:Os.7893.1.S1_at; gb|AK066658.1;	878	ref|NP_001063869.1|	2.00E-30	HIGH MOBILITY GROUP B 3
consensus:Rice:Os.536.1.S1_at; gb|AK099949.1;	1436	ref|NP_001042042.1|	3.00E-82	short-chain dehydrogenase/reductase (SDR) family protein
consensus:Rice:Os.27991.1.S1_at; gb|AK101995.1;	1745	ref|NP_001062946.1|	e-109	HISTONE ACETYLTRANSFERASE OF THE GNAT FAMILY 2

consensus:Rice:Os.19344.1.S1_at; gb|AK068184.1;	887	ref|NP_001043469.1|	e-31	unknown protein
consensus:Rice:Os.7698.1.S1_at; gb|AK121169.1;	744	ref|NP_001061171.1|	8.00E-58	ATG8C (AUTOPHAGY 8C); microtubule binding
consensus:Rice:Os.9100.1.S3_at; gb|AK068222.1;	5300	ref|NP_001119390.1|	0	binding
consensus:Rice:OsAffx.31710.1.S1_x_at; tigr|9640.m01173;	2199	ref|NP_201182.1|	0	DIACYLGLYCEROL KINASE 2
consensus:Rice:Os.48636.1.S1_at; gb|AK069638.1;	3449	ref|NP_001062293.1|		No hits above cut off
consensus:Rice:OsAffx.6894.1.S1_x_at; tigr|9638.m03344;	411	ref|NP_001065071.1|	2.00E-26	unknown protein
consensus:Rice:Os.24111.2.S1_at; gb|AK108209.1;	1051	ref|NP_001056519.1|	3.00E-33	unknown protein
consensus:Rice:Os.2613.1.S1_at; gb|AF309381.1;	1250	ref|NP_001066398.1|	3.00E-34	GLUTATHIONE S-TRANSFERASE 18
consensus:Rice:OsAffx.16824.1.S1_at; tigr|9636.m00365;	1556	ref|NP_001060962.1|	e-139	serine/threonine-specific protein kinase-like protein
consensus:Rice:Os.10930.1.S1_at; gb|BI806747;	551	ref|NP_001047483.1|	8.00E-33	phenylalanine ammonia-lyase
consensus:Rice:Os.25639.1.S1_at; gb|AK100844.1;	2227	ref|NP_001052535.1|	e-178	ROTAMASE FKBP 1
consensus:Rice:Os.8463.1.S2_at; gb|AK066026.1;	4160	ref|NP_001056347.1|	3.00E-88	kinase-related
consensus:Rice:Os.11668.1.S1_at; gb|AK073626.1;	991	ref|NP_001049891.1|	5.00E-60	TENA/THI-4 family protein
consensus:Rice:Os.40181.2.S1_s_at; gb|NM_191744.1;	597	ref|NP_001044060.1|	3.00E-83	AtRABG3a
consensus:Rice:Os.25486.1.S1_at; gb|AK103291.1;	2050	ref|NP_001044365.1|	2.00E-85	unknown protein
consensus:Rice:Os.25687.1.S1_x_at; gb|AK068993.1;	2498	ref|NP_001047484.1|	0	phenylalanine ammonia-lyase, putative
consensus:Rice:OsAffx.11501.1.S1_x_at; tigr|9629.m04531;	1614	ref|NP_001043779.1|	2.00E-62	exonuclease-related
consensus:Rice:Os.54493.1.S1_at; gb|AK105344.1;	1608	ref|NP_001057315.1|	e-155	calmodulin-binding heat-shock protein

consensus:Rice:Os.6756.1.S1_at; gb|AK072565.1;	1298	ref|NP_001062767.1|	9.00E-54	unknown protein
consensus:Rice:Os.32736.1.S1_at; gb|AK103085.1;	1229	ref|NP_001051453.1|	e-102	fatty acid hydroxylase-like protein
consensus:Rice:Os.12974.1.S1_at; gb|AK121422.1;	3231	ref|NP_001067910.1|	0	MCM2-related protein
consensus:Rice:Os.11407.1.S1_at; gb|AK106044.1;	2198	ref|NP_001054218.1|	e-142	ATPAO3 (POLYAMINE OXIDASE 3); oxidoreductase
consensus:Rice:Os.24751.1.S1_at; gb|AK071844.1;	1473	ref|NP_001059405.1|	5.00E-91	S-adenosylmethionine carrier
consensus:Rice:Os.7111.1.S1_x_at; gb|AK106228.1;	460	ref|NP_001047085.1|		No hits above cut off
consensus:Rice:Os.37571.1.S1_at; gb|AK063639.1;	1001	ref|NP_001062070.1|		No hits above cut off
consensus:Rice:Os.49084.1.S1_at; gb|AK064752.1;	1885	ref|NP_001057133.1|	e-166	putative NADH dehydrogenase
consensus:Rice:Os.27818.1.S1_at; gb|AK073491.1;	2110	ref|NP_001061433.1|	e-53	WRKY4 (WRKY DNA-binding protein 4); transcription factor
consensus:Rice:Os.25780.1.S1_s_at; gb|AK067751.1;	2640	ref|NP_001054078.1|	3.00E-50	zinc finger (C3HC4-type RING finger) family protein
consensus:Rice:Os.53107.1.S1_at; gb|AK070852.1;	1188	ref|NP_001046689.1|	3.00E-43	unknown protein
consensus:Rice:Os.4660.1.S1_s_at; gb|AK066494.1;	559	ref|NP_001049319.1|		No hits above cut off
consensus:Rice:OsAffx.26540.1.S1_s_at; tigr|9632.m04809;	588	ref|NP_001047768.1|	7.00E-24	RHA3B (RING-H2 finger A3B); protein binding / zinc ion binding
consensus:Rice:Os.8207.1.S1_at; gb|CB096959;	2696	ref|NP_001050457.1|	0	tRNA-splicing endonuclease positive effector-related
consensus:Rice:Os.10258.1.S1_at; gb|AK072446.1;	2890	ref|NP_001045208.1|	e-48	CPR5 (CONSTITUTIVE EXPRESSION OF PR GENES 5)
consensus:Rice:Os.5655.1.S1_at; gb|AK101293.1;	1387	ref|NP_001050402.1|	e-132	inosine-uridine preferring nucleoside hydrolase family protein
